# Retraction Note: Involvement of the glutamate/glutamine cycle and glutamate transporter GLT-1 in antidepressant-like effects of Xiao Yao san on chronically stressed mice

**DOI:** 10.1186/s12906-023-04089-3

**Published:** 2023-07-15

**Authors:** Xiu-Fang Ding, Yue-Hua Li, Jia-Xu Chen, Long-Ji Sun, Hai-Yan Jiao, Xin-Xin Wang, Yan Zhou

**Affiliations:** 1 School of Basic Medical Science, Beijing University of Chinese Medicine, Beijing, 100029 China; 2 Beijing Chaoyang Hospital, Capital Medical University, Beijing, 100043 China; 3 School of Basic Medicine, Henan University of TCM, Henan, Henan, 450046 China

**Retraction Note: BMC Complement Med Ther 17, 326 (2017)**

10.1186/s12906-017-1830-0

The Editor has retracted this article. After publication it was noted that in Fig. 6 there is overlap in Fig. 6A between the CA1 Model and XYS images and also in Fig. 6B between the CA1 Control and XYS images. The authors were asked to provide the raw data for the figure, however, the data provided by the authors could not be matched to the data used in the figure. The Editor has therefore lost confidence in the integrity of the data presented in this article.

None of the authors agree to this retraction.

